# Vestibular perceptual testing from lab to clinic: a review

**DOI:** 10.3389/fneur.2023.1265889

**Published:** 2023-10-04

**Authors:** Colin R. Grove, Brooke N. Klatt, Andrew R. Wagner, Eric R. Anson

**Affiliations:** ^1^Department of Otolaryngology Head and Neck Surgery, Johns Hopkins University School of Medicine, Baltimore, MD, United States; ^2^Division of Physical Therapy, Department of Physical Medicine and Rehabilitation School of Medicine, Emory University, Atlanta, GA, United States; ^3^Physical Therapy Department, University of Pittsburgh, Pittsburgh, PA, United States; ^4^Department of Otolaryngology—Head and Neck Surgery, Ohio State University Wexner Medical Center, Columbus, OH, United States; ^5^School of Health and Rehabilitation Sciences, Ohio State University, Columbus, OH, United States; ^6^Department of Otolaryngology, University of Rochester, Rochester, NY, United States; ^7^Physical Therapy Department, University of Rochester, Rochester, NY, United States; ^8^Department of Neuroscience, University of Rochester, Rochester, NY, United States

**Keywords:** vestibular, perception, spatial orientation, navigation, cognition

## Abstract

Not all dizziness presents as vertigo, suggesting other perceptual symptoms for individuals with vestibular disease. These non-specific perceptual complaints of dizziness have led to a recent resurgence in literature examining vestibular perceptual testing with the aim to enhance clinical diagnostics and therapeutics. Recent evidence supports incorporating rehabilitation methods to retrain vestibular perception. This review describes the current field of vestibular perceptual testing from scientific laboratory techniques that may not be clinic friendly to some low-tech options that may be more clinic friendly. Limitations are highlighted suggesting directions for additional research.

## 1. Introduction

Self-motion perception, the conscious awareness of active or passive motion/orientation of one’s body in space, is influenced by multiple factors in health and disease, including the state of peripheral vestibular reflexes (1), central postural control and spatial orientation systems (2, 3), attentional networks (4), emotional states (5), and behavioral responses to perceived postural threats (6). Individuals with persistent complaints of vertigo/disequilibrium experience abnormal vestibular self-motion perception and spatial orientation (7–12). Subjective experiences and reported symptoms are often incongruent with the results of routine diagnostic tests of vestibular function and vary across individuals with similar diagnoses (13–20). This can be particularly troublesome for individuals with dizziness who present with normal vestibular function testing which can be the case for vestibular migraine, mal de debarquement syndrome, and persistent perceptual postural dizziness (21–23). It is interesting to note that several methods of vestibular perceptual testing are able to distinguish healthy individuals from individuals with vestibular disease (8, 24–29). Performance on some vestibular perception tests correlates with balance ability (30–32), which suggests clinical utility (33). However, the role of vestibular perceptual testing in the diagnosis and treatment of individuals with dizziness remains undefined, possibly in part due to the specialized equipment (34) or time involved in testing (35). In this review, we synthesize the existing literature describing several different forms of vestibular perceptual testing that range in application from the laboratory setting to clinical use.

We expand on the traditionally narrow definition of vestibular perception (movement detection or movement discrimination) to include spatial orientation, spatial navigation, and spatial cognition as vestibular signals are known to contribute to more global spatial perception\cognition and navigation (1, 36–44). In the first section, we briefly review the anatomical pathways contributing to vestibular perceptual abilities as well as review the historical and state of the art approaches to vestibular self-motion perception. In the second section, we review approaches to vestibular spatial orientation and verticality. In the third section, we review approaches to vestibular spatial navigation. In the fourth section, we review global perspectives on vestibular cognition. We conclude the review with the authors’ perspectives on clinical value and clinical utility as well as some potential recommendations for the future.

## 2. Vestibular perception: anatomy to laboratory testing

Vestibular perceptual thresholds are a laboratory-based technique used to measure an individual’s sensitivity to passive self-motion cues (33, 45). This is accomplished by measuring the ability to perceive a passive whole-body motion stimulus in the absence of visual feedback (e.g., blindfolded or in a light tight room) and with tactile and auditory cues minimized (e.g., noise cancelling headphones and padded seats). Although the specific test procedures and statistical approach used to estimate a threshold will ultimately determine the exact interpretation of the value reported, in general, an individual with a lower vestibular threshold should, on average, be able to reliably perceive smaller motion stimuli. A person’s “vestibular threshold” therefore serves as a behavioral measure of the sensitivity of the vestibular system to a specific vestibular motion stimulus (e.g., tilt, rotation, or translation). In this review we will briefly cover the methodology used to measure vestibular thresholds, and then provide an overview of normal and pathologic responses captured by vestibular thresholds. Interested readers are referred to prior, more comprehensive reviews that cover the experimental methods and statistical approach to estimating vestibular thresholds (33, 45–48).

The most common method for measuring vestibular thresholds is a direction recognition task (DRT) (45). In a DRT, an individual is passively moved and then asked to judge the direction of the motion stimulus (e.g., left vs. right or up vs. down). Although this is commonly done using a two-alternative forced choice task (e.g., deciding between a left and right yaw rotation), recent approaches have successfully used up to 12 alternative choices (6 motion planes and 2 directions for each) (35, 49). An adaptive staircase procedure — decreasing the size of the motion stimulus after a number of consecutive correct responses and increasing the size of the stimulus after an incorrect response (50) — is also typically used to efficiently sample stimulus values around an individual’s threshold (45). The vestibular threshold is then determined from these binary responses by taking either (a) the average of the terminal staircase reversals [e.g., averaging the stimuli at the step up (after an incorrect response) and the step down (after a number of correct responses) (7, 51, 52) or (b) by fitting the data to a psychometric function (30, 47, 53–56). Fitting the data to a psychometric function holds the advantage of using all of the participant’s responses, rather than only the final number of reversals, and also yields a threshold value with a clear physiological interpretation based upon signal detection theory (45, 57, 58). The methods described by Merfeld and colleagues (45, 46, 48) involve fitting the binary subject responses (e.g., right versus left) and stimulus magnitudes to a Gaussian cumulative distribution function (CDF) to estimate the 1σ (i.e., “one sigma”) threshold. This parameter corresponds to the stimulus magnitude that would on average be perceived accurately on ~84.1% of trials. Therefore, if a human observer has a yaw vestibular threshold of 0.5°/s, assuming zero bias, this individual should on average be able to accurately perceive the direction (e.g., right vs. left) of a 0.5°/s yaw rotation on 84.1% of trials. For an individual that instead has a threshold of 5°/s — putatively resulting from a bilateral vestibular lesion — the probability of correctly sensing the direction of the same 0.5°/s motion stimulus is nearly the same as the flip of a coin (~53%).

Based upon principles of signal detection theory, the 1σ parameter is equivalent to the width of the CDF, and as a result, the threshold parameter is proportional to the noise in an individual’s perceptual estimate of the motion stimulus [see (45, 59, 60) for additional details on vestibular noise]. Vestibular thresholds can therefore describe either “sensory noise,” or “sensory precision,” with the two terms being inversely related to the threshold parameter — higher thresholds indicating increased noise and lower precision (59). Regardless of whether the threshold is interpreted as a measure of “sensitivity” or a measure of “sensory precision/noise,” the definitions are harmonious. The threshold parameter in actuality reflects the ratio between signal strength and sensory noise (i.e., the signal to noise ratio). As such, a lower signal to noise ratio, as represented by a higher threshold, will make the relevant motion stimulus harder to separate from internal sensory noise resulting in both (a) the need for a larger motion stimulus to permit an accurate perception of motion (*lower sensitivity*) and (b) greater variability in the subjective experience of the motion (*imprecision or increased noise*) [see (59) for additional reading on this topic]. It is also worth mentioning that from these methods, a “bias” parameter that describes the mean of the CDF (i.e., the displacement of the CDF along the abscissa) is also estimated (45). However, in comparison to thresholds, vestibular bias has yet to be studied in-depth in patient populations, and as a result we focus the present review only on the threshold parameter.

**Figure 1:**
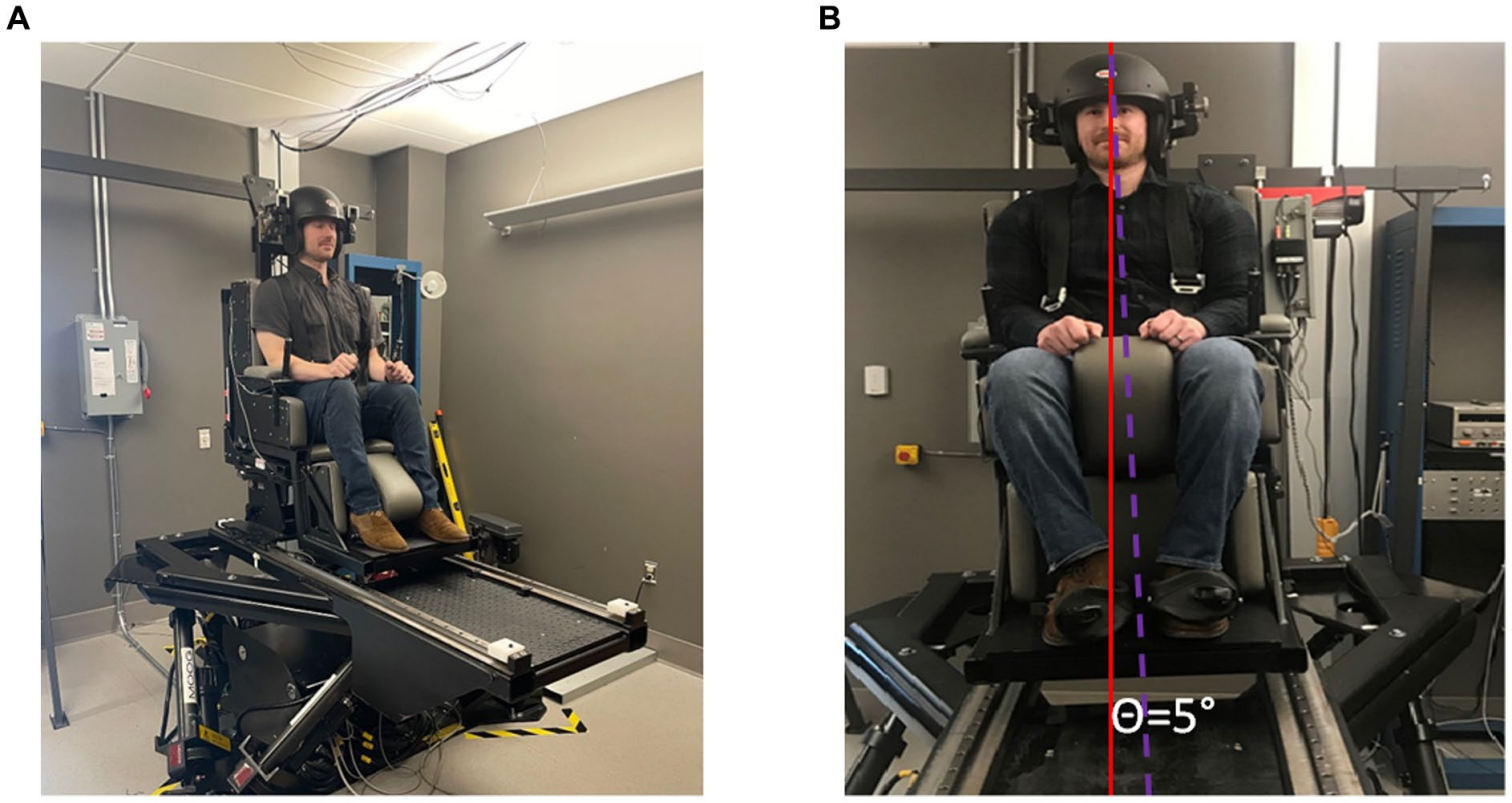
**(A)** Exemplar image of a subject sitting in a chair mounted to a hexapod base participating in an experiment to quantify roll-tilt threshold. Typically, this experiment would be conducted in the dark to emphasize vestibular signals of inertial motion. Subjects respond with a button press to indicate whether they tilted to the right or the left. **(B)** In this example the subject is tilted 5° to the right. The solid red line indicates earth vertical and the dashed purple line indicates body orientation after a head centered roll-tilt to the right.

Throughout this section of the review, the term “vestibular threshold” will also be more precisely specified based upon the motion trajectory (translation, rotation, or tilt), plane of motion (e.g., fore-aft, interaural, superior–inferior) and the frequency of the motion stimulus; since single cycles of sinusoidal acceleration are most often used as the motion stimulus, the stimulus frequency describes the inverse of the motion duration (e.g., 1 Hz = 1 s per motion, 0.2 Hz = 5 s per motion). The vestibular system is multi-modal, with five sensors within each labyrinth that collectively allow us to sense and response to rotations (via the semicircular canals), translations (via the otolith organs), and tilts (via a combination of canal and otolith inputs) of the head (61). The relative excitability of afferent neurons within the different end-organs is determined primarily by the type of motion (rotation versus translation) and frequency of the motion stimulus (61). As such, by carefully selecting the plane of motion and the stimulus frequency (30, 53, 56, 62), vestibular thresholds can preferentially quantify motion perception with predominant contributions from the different end-organ pairs, see Figure 1 for an example. Table 1 summarizes several motion profiles commonly used to assay vestibular perceptual thresholds with predominant contributions from the various end-organs. This list is not all inclusive, but is instead based upon a recent series of empirical studies (53, 56, 62–64).

**Table 1:** Common motion profiles used to measure vestibular perception with predominant contributions from the different end organs.

Vestibular end-organ	Motion(s)	References
Lateral canals	Earth vertical yaw rotations	(51, 62, 63)
Vertical canals	Earth horizontal tilts (≥2 Hz*) earth vertical rotations	(56, 62–64)
Utricles	Interaural translations (<2 Hz**) quasi-static tilts	(24, 53, 62–64)
Saccules	Superior–inferior translations (<2 Hz**) quasi-static tilts	(53, 62, 63)
Canal-otolith integration	Earth horizontal tilts (<1 Hz***)	(56, 62–64)

*2 Hz earth-horizontal tilts thresholds were shown to be equivalent to 2 Hz earth-vertical rotation thresholds, supporting the predominant use of vertical canal signals. **Relative to 1 Hz translations, translations at 2 Hz were shown to be more influenced by extra-vestibular (e.g., proprioceptive) motion cues. ***Thresholds for earth horizontal tilts improve at lower frequencies (<1 Hz), presumably due to the integration of otolith and vertical canal inputs.

### 2.1. Normative data and age effects

Bermúdez Rey et al. measured vestibular thresholds in 105 asymptomatic adults between 18 and 80 years of age (30). Direction recognition thresholds were measured for 1 Hz yaw rotations (lateral canal), 1 Hz interaural translations (utricles), 1 Hz superior–inferior translations (saccules), and roll tilt at 2 frequencies (1 Hz – vertical canals, 0.5 Hz – canal-otolith integration). A linear increase in each of the thresholds was observed beginning at approximately age 40 (30). The strongest effect seen was for superior–inferior thresholds, with an observed increase of 83% per decade (Table 1). However, by comparison yaw rotation thresholds were relatively spared, showing only a weak association with age (15% increase per decade). Although the study by Bermúdez Rey et al. is the largest to date, additional studies of smaller sample sizes similarly support (1) an age effect on vestibular translation thresholds, specifically interaural translation and superior–inferior translation thresholds (29, 54, 65), as well as (2) a weak association between age and yaw rotation thresholds (54, 66, 67). Only a single study (*N* = 28) showed a lack of an association between interaural threshold and age (68), however the study by Kingma and colleagues showed an effect of age on fore-aft translation thresholds and the study only included adults up to age 60, potentially reducing the strength of the age effect on interaural translation thresholds. This is consistent with epidemiology reports indicating reduced otolith function in the 7th and 8th decade (69).

Gabriel et al. recently used a two-interval detection task (“did you move in interval 1 or 2”) and a two-interval magnitude discrimination task (e.g., “was movement 1 or 2 larger”) to determine differences in 0.5 Hz superior–inferior translation and 0.5 Hz pitch tilt thresholds between young adult (*N* = 18) and older adult (*N* = 19) participants (70). They found that older adults demonstrated an increase in detection thresholds, but not discrimination thresholds, relative to young adults (70). This study also found that within the older adult group, superior–inferior detection thresholds, pitch detection thresholds, and pitch discrimination thresholds each showed significant correlations with quiet stance postural sway (70). Bermúdez Rey and colleagues also compared vestibular thresholds to “pass/fail” balance performance on the four conditions of the Modified Romberg Balance Test. In adults over the age 40 (*N* = 56), they showed that an individual’s 0.2 Hz roll tilt threshold (a test requiring the central integration of canal and otolith signals) was the strongest predictor of whether they were able to complete an “eyes closed, on foam” balance test (30, 31). This relationship between roll tilt thresholds and postural control has since been further characterized by studies showing significant correlations between roll tilt thresholds and quantitative measures of postural sway (32, 71). The specific link between roll tilt thresholds and postural control does however contrast with the findings of Gabriel et al. (70), as the study by Bermúdez Rey and colleagues did not find a significant association between balance performance and superior–inferior translation thresholds (30, 31, 72). Yet, the disparate methods used (two-interval detection and discrimination tasks versus a one-interval direction recognition task) prevents a direct comparison between the findings of the two studies.

**Figure 2:**
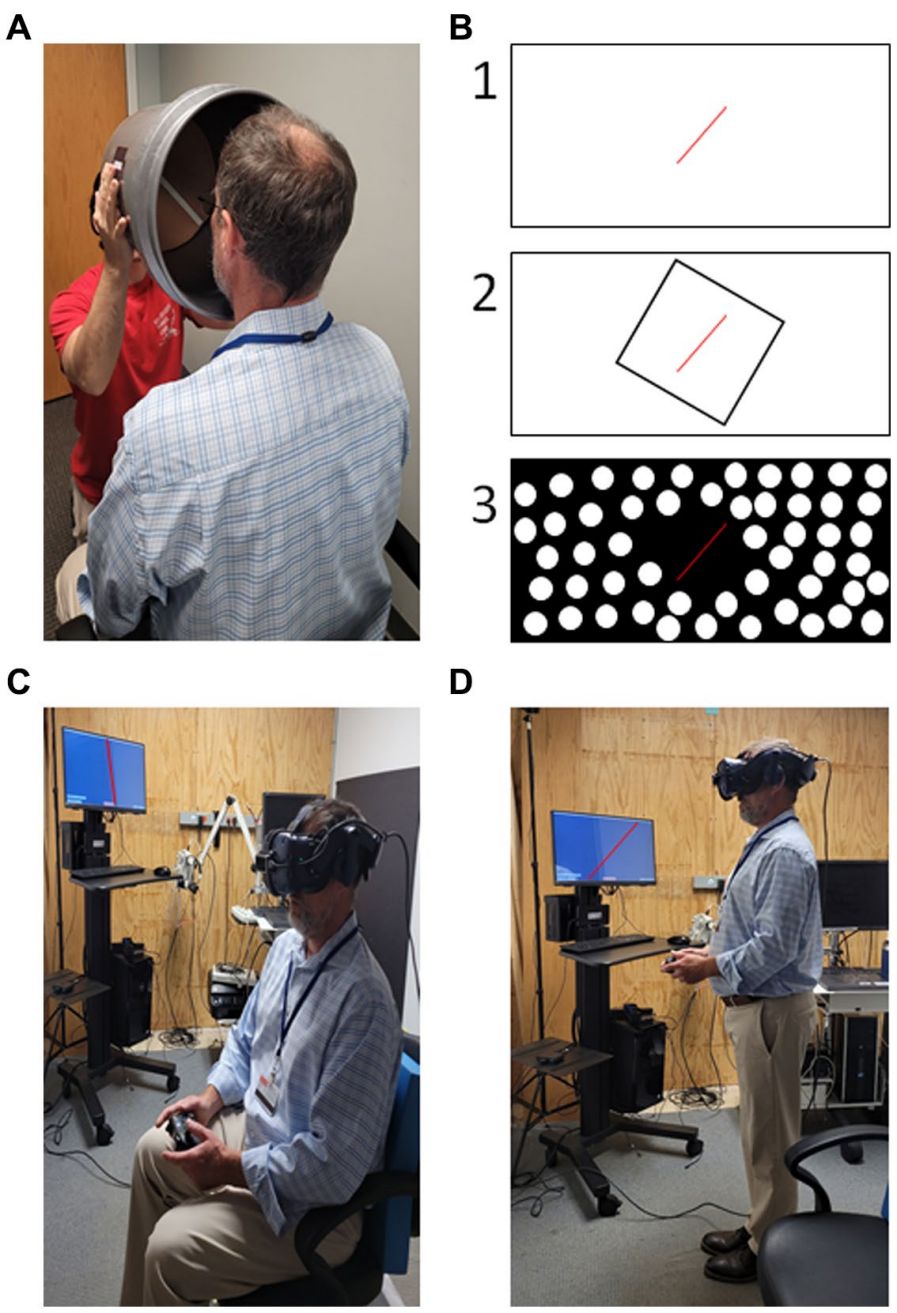
Examples of subjective visual vertical (SVV) testing. **(A)** Low-tech “bucket” method testing a seated subject (73). **(B)** Example styles of high-tech SVV testing with projection screens or using virtual reality, (1) tilted line on blank background; (2) “Rod and Frame” - tilted line presented in tilted square; (3) dynamic SVV – background of dots rotating clockwise or counter-clockwise with a tilted line in the center. **(C)** SVV performed in virtual reality while seated. **(D)** SVV performed in virtual reality while standing. Patient task in all scenarios is to indicate when the visible line is oriented with vertical either verbally or by button press. Photos in **(C,D)** digitally altered to improve visibility of the tilted red line presented on the computer monitor.

**Figure 3:**
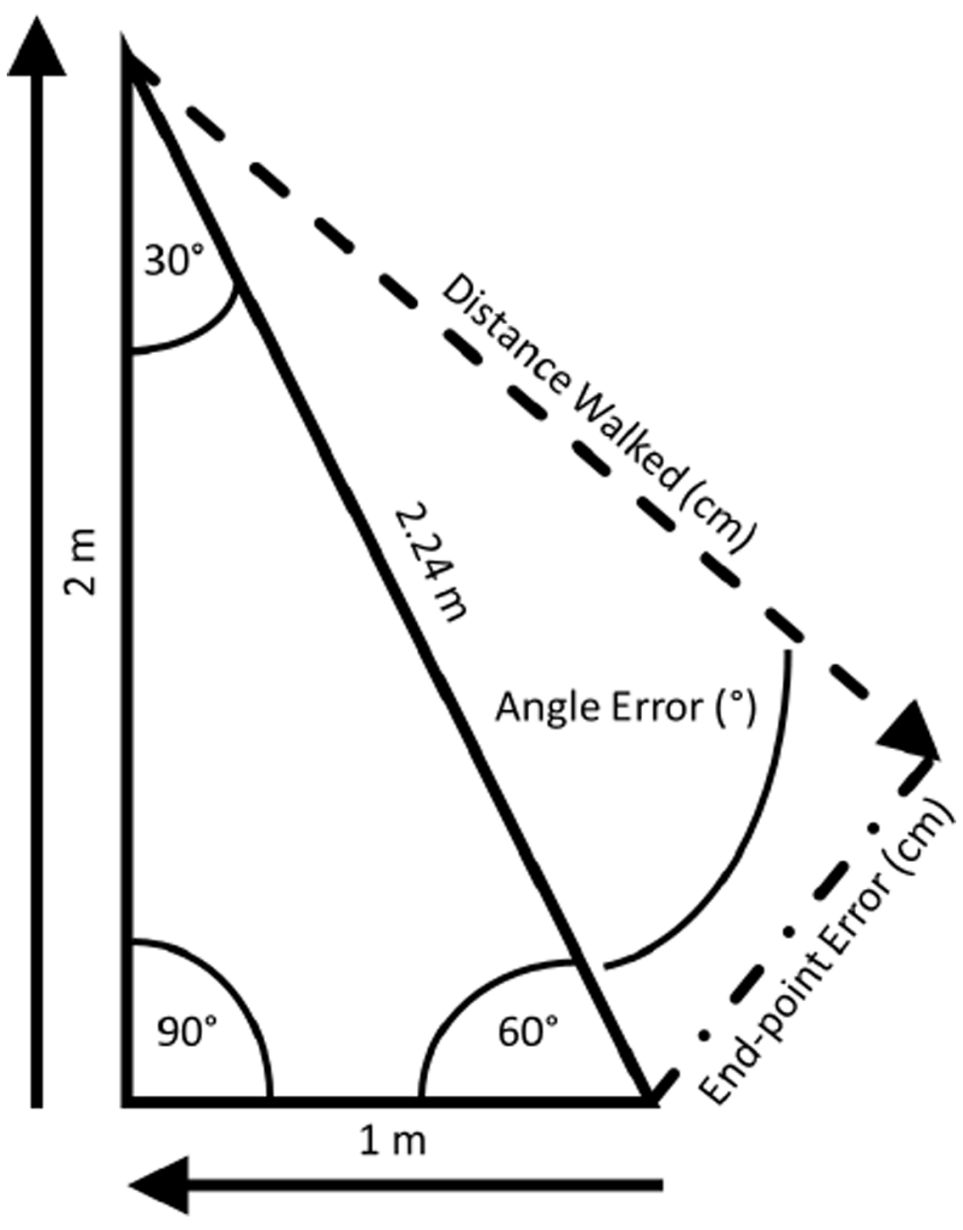
The triangle completion test (TCT): the TCT (74) is conducted using right triangles, the sides of which are distinctly marked on the floor. Participants don a blindfold and noise-attenuating headphones and, then, are instructed to visualize the triangle to be walked. Participants are passively guided along one leg, a 90° turn, and then the second leg. Once the examiner lets go, participants are instructed to “turn and complete the triangle”.

As it represents the largest normative dataset currently available, Table 2 provides an overview of the threshold values per decade measured in the study by Bermúdez Rey et al. (30). Despite the healthy nature of the population [see (30) for exclusionary criteria used] the thresholds measured displayed considerable variability within the cohort (30) (Figures 1–3). The source of variability in thresholds between asymptomatic adults may be indicative of subclinical changes to the vestibular system that cause an increases in thresholds prior to the onset of symptoms (60, 72) or may reflect the influence of confounding factors, unaccounted for in the current threshold assessment protocols. This remains an open area of investigation.

**Table 2:** Data presented show the geometric mean and 95% confidence intervals for each threshold measure for each age range (*N* = 105).

	Yaw rotation	Interaural translation	Superior–inferior translation	Roll tilt	Roll tilt
Stimulus frequency	1 Hz (1 s/cycle)	1 Hz (1 s/cycle)	1 Hz (1 s/cycle)	0.2 Hz (5 s/cycle)	1 Hz (1 s/cycle)
18–29	1.06 (0.87–1.28)	0.61 (0.48–0.79)	1.36 (1.04–1.77)	0.37 (0.31–0.44)	0.70 (0.60–0.82)
30–39	1.04 (0.86–1.26)	0.64 (0.52–0.78)	1.26 (0.96–1.67)	0.37 (0.30–0.46)	0.65 (0.52–0.81)
40–49	0.99 (0.83–1.19)	0.79 (0.59–1.05)	1.91 (1.44–2.53)	0.46 (0.37–0.59)	0.92 (0.71–1.18)
50–59	1.16 (0.94–1.44)	0.99 (0.75–1.29)	2.81 (2.23–3.53)	0.57 (0.45–0.72)	1.19 (1.00–1.42)
60–80	1.45 (1.14–1.84)	1.15 (0.87–1.53)	4.35 (2.86–6.60)	0.67 (0.51–0.88)	1.74 (1.29–2.35)
Change per decade	15%	46%	83%	32%	56%

The percent change per decade is relative to a baseline, under which no age effects were seen. This table is recreated from (30) with permission.

### 2.2. Vestibular hypofunction

Several studies have characterized the influence of peripheral vestibular hypofunction on vestibular thresholds. Due to the time required to complete a standard staircase procedure (i.e., to 50 to 200 trials over 8 to 30 min) (47), Cutfield et al. developed an abbreviated ‘time to response’ threshold task for the assessment of patients in the acute stages of vestibular neuritis (VN) (75). In contrast to the two-alternative forced choice task described above, individuals are asked to report the perceived direction of motion as quickly as possible, with the threshold being defined by the latency between the motion onset and a button press. Cutfield et al. (75) showed that patients with acute unilateral vestibular hypofunction (UVH) due to VN (*N* = 12) showed a significant elevation in yaw rotation thresholds, as indicated by a prolonged duration between motion onset and subject response. In a follow-up study, Cousins et al. similarly showed that individuals with acute UVH (*N* = 25) due to VN showed an elevation of vestibular thresholds relative to healthy controls (*N* = 30) within the first 1–5 days from symptom onset (76). Rotation thresholds were increased for both ipsilesional and contralesional rotations but showed a greater elevation toward the lesioned ear. Even after a period of presumed vestibular compensation, participants showed a consistent elevation in thresholds at 10 weeks from the time of lesion (76). Recently, Madhani et al. used a two-alternative forced choice task to measure yaw rotation thresholds in a group of 8 participants diagnosed with neurofibromatosis (NF-2) related schwannomatosis (*N* = 5 bilateral and *N* = 3 unilateral) and 38 participants with sporadic unilateral vestibular schwannomas (SWN) (77). They found that 1 Hz yaw rotation thresholds were elevated for both groups relative to healthy controls (*N* = 23) (77). In two of the patients with bilateral NF2-SWN, perceptual thresholds were also improved following treatment with chemotherapy (bevacizumab) (77). Since the healthy labyrinth provides a bidirectional response to yaw rotations secondary to an inhibitory response from the contralateral side, approaches such as those utilized by Roditi and Crane (54) that use independent staircases for right and left rotations may be of benefit in the assessment of vestibular hypofunction.

Prior to discussing the literature describing the impact of bilateral vestibular lesions on vestibular thresholds, a distinction must first be made between studies that include “bilateral vestibular hypofunction” (BVH) and studies that have intentionally recruited patients with a total bilateral vestibular loss due to bilateral vestibular labyrinthectomies (BVL). In BVH, the vestibular apparatus remains present, and as a result BVH is often characterized by an incomplete loss of vestibular responses bilaterally. Individuals who have instead experienced a bilateral labyrinthectomy lack a vestibular system, and as such, produce no vestibular response to motion. As a result, studies that include individuals with BVL have primarily been conducted as a means to describe vestibular contributions to motion perception (62, 63), rather than as a means to gain specific insights into a common patient population. Valko et al. found that patients with BVL (*N* = 3) had vestibular thresholds that were between 1.3 and 56.8 times greater than age-matched healthy controls (*N* = 14) with the magnitude of the difference depending upon both the specific motion direction (interaural translation, superior–inferior translation, yaw rotation, and roll tilt) and frequency (62). In a more recent study, Kobel and colleagues showed that vestibular thresholds for 2 individuals with BVL were between 2 and 35 times greater than healthy controls for a more comprehensive set of motions that included roll tilts (head rotated about an earth horizontal axis while sitting upright) and roll rotations (rotated about an earth vertical axis while supine) performed across a broad range of frequencies (0.2 to 2 Hz) (63). Together these studies highlight the dominance of vestibular inputs during vestibular thresholds assayed using a direction recognition task.

In individuals with BVH, early studies looking at motion perception were less conclusive. In the late 1990’s and early 2000’s, two studies that included four and five individuals with BVH respectively, found a lack of evidence to support a difference in translation (78) or tilt (79) perception relative to healthy adults. However later, Priesol et al. captured a more comprehensive battery of vestibular thresholds in four individuals that each had received a prior diagnosis of idiopathic BVH (26). They found that thresholds were not uniformly elevated across this sample, but instead individuals with BVH showed a selective increase in yaw rotation thresholds (assaying the lateral canals) and low frequency interaural translation thresholds (assaying the utricles) (26). Valko et al. similarly found a larger difference in translation thresholds at lower frequencies in individuals with BVL which suggests a potential increase in the contributions of non-vestibular inputs at the higher frequencies (62). Priesol et al. did not however show a significant difference between individuals with BVH and healthy controls for superior–inferior translation (assaying the saccules) or roll tilt (assaying central canal-otolith integration) thresholds. More recently, Shayman and colleagues did find a significant increase in 1 Hz yaw rotation thresholds for individuals with BVH (80), however this study included only 3 participants, and thus differences in the results of Priesol (*n* = 4) and Shayman (*N* = 3) may be a result of the small sample sizes. Since vestibular thresholds vary considerably between even healthy (asymptomatic) individuals (Table 2), the results of studies that include small sample sizes should be viewed with caution.

In two larger studies of adults with BVH (*N* > 30), van Stiphout et al. and Agrawal et al. found more global changes in vestibular thresholds (49, 65). Van Stiphout and colleagues measured thresholds for 3 planes of translation (fore-aft, interaural, and superior–inferior) and 3 planes of rotation/tilt (yaw rotation, pitch tilt, and roll tilt) in 37 adults with BVH and 34 healthy controls. In contrast to a standard two-alternative forced choice task, a 12 alternative choice task was used that required the participants to select both the direction (e.g., right vs. left, up vs. down) and type of motion (rotation vs. translation) experienced. The data were then analyzed within two subgroups, a middle-aged group aged 40–59 (*N* = 18 with BVH) and an older adult group aged 60–79 (*N* = 19 with BVH). With the exception of superior–inferior translation thresholds in the older adult group and roll tilt thresholds in the middle-aged group, all other thresholds measured were significantly elevated relative to age-matched healthy controls. Of note, superior–inferior translation thresholds are markedly increased in asymptomatic older adults (30) (Table 2), and thus, this may explain the lack of an effect of BVH of superior–inferior thresholds specifically in the older adult group. An additional study by Agrawal and colleagues found significant increases in interaural, fore-aft, and superior–inferior thresholds in individuals with BVH (*N* = 33) relative to healthy controls (*N* = 42) (65).

The principal take away from these studies is that vestibular thresholds are capable of detecting specific changes in vestibular self-motion perception in individuals with peripheral vestibular hypofunction. Since vestibular hypofunction may include varying levels of damage to the different branches of the vestibular nerve or to the individual vestibular end-organs, a characteristic change in a specific motion paradigm should not be assumed. Instead, these data highlight the need for future studies to characterize a comprehensive battery of threshold measures to fully capture the impact of UVH and BVH on the vestibular system.

### 2.3. Vestibular migraine and Meniere’s disease

Over the past decade, vestibular thresholds have been studied as a potential objective measure to help with the diagnosis of vestibular migraine (VM). Lewis et al. showed that individuals with VM (*N* = 8) demonstrated significantly lower 0.1 Hz roll tilt thresholds compared to both healthy controls (*N* = 8) and individuals with non-vertiginous migraine (*N* = 8) (7). Alternative vestibular thresholds that relied primarily upon either the semicircular canals (1 Hz dynamic roll tilt, 0.1 Hz supine roll rotation, and 1 Hz supine roll rotation) or the otoliths (quasi-static roll tilt) in isolation did not differ between patients with VM and the other groups. In a follow-up study, King et al. showed that individuals with VM (*N* = 12) displayed low to mid frequency (0.05 to 0.2 Hz) roll tilt thresholds that were significantly lower compared to individuals with non-vertiginous migraine (*N* = 12), healthy controls (*N* = 12), and individuals diagnosed with Meniere’s disease (MD; *N* = 8; only 0.2 Hz roll tilt thresholds were measured in this group) (24). Similar to the earlier study, thresholds for higher frequencies of roll tilt (≥1 Hz) as well interaural translations (0.2, 0.3, and 0.5 Hz) and supine roll rotations (0.2 and 0.5 Hz) were not significantly different between the individuals with VM and either the healthy controls or non-vertiginous migraineurs.

Due to the overlapping clinical presentations of VM and MD, additional studies have also attempted to use vestibular thresholds to differentiate between these two patient groups. Bremova et al. found that patients with MD (*N* = 27) demonstrated higher 1 Hz interaural, fore-aft, and superior–inferior translation thresholds compared to individuals with VM (*N* = 20); only superior–inferior and fore-aft thresholds were elevated relative to the healthy control group (*N* = 34) (29). Consistent with the findings of Lewis et al. (7) and King et al. (24), individuals with VM did not display differences in translation thresholds relative to healthy controls (29). Using a “time to response” task, Bednarczuk et al. did however find that patients with VM (*N* = 15) showed a longer response latency during earth vertical yaw rotations when compared to healthy controls (*N* = 15), as well as compared to individuals with non-vertiginous migraine (*N* = 15) (81). A group with benign paroxysmal positional vertigo (BPPV, *N* = 15) was also included and showed similar yaw rotation thresholds as the VM group (81). It is worth highlighting that this timed response task differs substantially from the forced choice direction recognition task used in the prior studies of individuals with VM.

From these data it can be concluded that vestibular thresholds in individuals with MD are consistent with a loss of sensitivity to self-motion cues, analogous to the effect of vestibular hypofunction. Conversely, individuals with VM show a higher sensitivity to low to mid frequency roll tilts (i.e., lower thresholds). Since the perception of a low to mid frequency roll tilt stimulus requires that the brain perform computations that combine angular velocity signals from the vertical semicircular canals with gravitoinertial acceleration signals from the otolith organs [see (82–85) for greater details], these findings in individuals with VM have been posited to reflect abnormal central canal-otolith integration (7, 8, 24).

### 2.4. Traumatic brain injury

A traumatic brain injury (TBI) can be readily diagnosed by correlating symptom onset with a traumatic event. However, recently considerable attention has been given to further classifying individuals into concussion subtypes (e.g., vestibular, headache, cognitive) to enable early access to targeted medical and rehabilitative services (86, 87). Vestibular dysfunction following TBI is primarily determined by the subjective report of symptoms elicited by specific head motions (88); physiologic vestibular metrics that capture vestibular impairments have been more elusive (89). A recent study found that vestibular perceptual thresholds may aide in the identification TBI related vestibular impairment, referred to as “vestibular agnosia” (44). Seemungal and colleagues showed that in the acute phase after a TBI (*N* = 37), yaw rotation thresholds were significantly elevated relative to age-matched healthy controls (*N* = 37) (44). Individuals with elevated vestibular thresholds also showed an increase in postural sway (44), increasing the likelihood that the changes observed represented a change in vestibular function. However, the isolated assessment of yaw rotation thresholds restricts these data to describing only the influence of TBI on the processing of lateral canal signals, and the broader impact of TBI on the remaining vestibular modalities requires further study.

### 2.5. Benefits of vestibular thresholds

Vestibular thresholds are not currently available as a clinical assessment for individuals with suspected vestibular pathology. Nevertheless, thresholds hold several notable advantages over the currently available vestibular clinical assessments. One of the primary advantages to vestibular thresholds is the ability to quantify different vestibular modalities using a single experimental methodology. While vestibular thresholds are not free of confounding influences (e.g., attention, fatigue) these factors should similarly influence the perception of different motion paradigms (tilt, translation, or rotation thresholds) and as such, non-vestibular factors should not exert a disparate influence on translation versus rotation thresholds. By comparison, standard clinical metrics assess the semicircular canals by quantifying the vestibulo-ocular reflex (e.g., calorics, video head impulse testing) and the otolith organs by measuring vestibular evoked myogenic potentials (VEMPs). The marked differences in experimental methodologies (oculomotor versus electromyographic responses) introduces the potential for confounding factors that may have disparate influences on the specific canal or otolith assessments. The second fundamental advantage is that vestibular thresholds are the most direct method for specifically measuring vestibular sensation in human participants. Alternative vestibular assessments require that sensory dysfunction be inferred from changes in the sensorimotor response observed (e.g., increased sway, decreased slow phase eye velocity, absent myogenic potentials), and as a result, these measures represent changes in sensory function alongside a sensorimotor transformation and the integrity of the motor limb of the response.

### 2.6. Limitations of vestibular thresholds

Vestibular thresholds possess several limitations that may serve as a barrier to their eventual clinical implementation. There is currently an absence of standardized methodologies for measuring and analyzing thresholds. As a result, data collected in different laboratories should be compared with caution. An additional barrier to the use of thresholds as a diagnostic measure is the variability in thresholds within samples of healthy, asymptomatic adults. Since thresholds can vary widely in healthy adult populations (30), identifying cut-off values for vestibular pathology is challenging. It is however likely that this variability is a result of both natural variance within the population as well as variability inherent to the threshold assessment paradigms currently used. The standardization of methods, and the identification of “normative” data and cut-off values for vestibular pathology are critical steps needed to develop vestibular thresholds as a viable clinical assessment tool. Finally, the assessment of thresholds currently requires specialized equipment and software that are not at the present time available in clinical settings. It is however worth highlighting that the cost of a 6-degree-of-freedom motion platform is comparable to the cost of the computerized dynamic posturography platforms that are commonly found in many audiologic and physical therapy clinics. As such, if the clinical utility of vestibular thresholds can be established, this final limitation is unlikely to pose a substantial challenge to the clinical implementation of vestibular thresholds.

## 3. Vestibular perception: spatial orientation and verticality

Moving beyond detection and direction discrimination of self-movement, we next focus on vestibular contributions to spatial perception. Spatial orientation can be conceptualized as the ongoing estimate of the three-dimensional spatial relationship between self and the surrounding world which necessarily implies sensory integration (90–93), and one’s ability to update the spatial reference frame of self-relative to the world after moving within the world (94–97). This process depends on successful and accurate integration of egocentric (vision, vestibular, somatosensation) self-motion cues and allocentric spatial representations, such as landmarks (2, 98, 99). Not surprisingly, spatial orientation involves distributed higher cognitive processing (43, 100–104), and can be adversely impacted by cognitive impairment, stress, and mental health disorders (5, 105–107). For the purpose of this review, we will restrict this topic to vestibular contributions to spatial orientation: the otoliths and semicircular canals (37, 39, 95, 108–110). We also distinguish spatial orientation (rotation only) from directional linear translations (heading) that may be navigation specific. Spatial navigation will be discussed in the next section.

Rotational vestibular signals directly contribute to three-dimensional compass mapping of space (111, 112). Specifically semicircular canal inputs provide input to head direction cells via thalamic pathways (39, 113, 114), thought to directly contribute to our ability to recognize which direction we are currently facing. This head in space directional information combined with the inertial signal from motion allows for recognition of which direction we were previously facing as well as updating to the new spatial orientation (1, 28, 101, 108). Similar to frequency dependent non-linearity observed with the vestibulo-ocular reflex, spatial orientation is worse at low frequencies (99). Importantly, spatial updating depends on knowledge of results of the movement based on integrating sensory feedback or cognitive knowledge (108), and greater reliance on cognitive representations of space is needed when individuals are asked to delay their response rather than immediately respond (115). Rotational spatial orientation can be conceptualized based on the response question: “where\what am I currently facing” (1, 28, 108) versus “where\what was I facing” (99, 116). Responses vary by metrics from reporting degrees of rotation, analog clock positions, or allocentric world fixed objects as proxies for rotation.

### 3.1. Normative data and age effects

For small low frequency rotations (~16°) reorienting errors (“where were you facing”) in healthy subjects are biased in the direction of physical rotation (exaggerating displacement) but normalized at higher frequency rotations (99). For larger rotations, average errors in healthy adults are relatively small ~25° or less (1, 28, 108). There is conflicting information regarding the potential for an aging effect on spatial orientation updating. Jáuregui-Renaud initially found no relationship between age and spatial orientation updating ability (28). However, using a similar paradigm, we demonstrated an aging effect such that older individuals made larger errors than younger individuals independent of vestibular function (108). In a study that involved self-controlled rotation back to the origin following a rotation in the dark, Zachou and Bronstein identified an age-related decline in accuracy for the return to origin, but not for the more complex complete the circle condition (97). Importantly, this style of spatial orientation testing (rotational trajectory matching) may involve different cognitive processes and has the potential for increased sensory noise associated with aging to negatively impact accuracy on the spatial estimate for both the initial rotation and the return rotation (59). Taken together, these findings suggest that increased age has a negative influence on spatial orientation for relatively simple spatial orientation tasks, which may reflect the reduction in vestibular function associated with loss of hair cells and afferent neurons known to occur with aging (69, 117, 118). The lack of an aging effect for more complex tasks may indicate greater resilience to aging effects associated with higher cognitive processes that estimate space or time (119–121).

#### 3.1.1. Vestibular hypofunction

Individuals with BVH demonstrate larger spatial errors during rotational testing compared to healthy individuals, tending towards underestimation for large amplitude spatial rotation tests (1, 28). Interestingly, individuals with UVH perform with similar spatial accuracy compared with healthy individuals, regardless of presence of dizziness. Intact vestibular function is linked to the ability to represent external space as well as accurate body spatial schema (122–124). This may explain why individuals with unilateral vestibular loss perform at levels similar to healthy individuals. The existing spatial orientation tests are performed at peak velocities below inhibitory cut-off, allowing afferent rotational signals from the intact ear to provide sufficient self-motion signals for spatial integration (125). In this context, any residual function should be sufficient to perform supra-threshold spatial orientation tasks with relative accuracy.

### 3.2. Verticality perception

The related concept of verticality (gravitational) perception can be evaluated using several different frameworks. Arguably the most common clinical method of verticality perception is subjective visual vertical (SVV) (126–129); although, subjective visual horizontal testing can also be examined (73, 130, 131). Visual vertical/horizontal perception involves orienting or judging a line with respect to the perceived direction of gravity (or orthogonal to gravity in the case of horizontal estimates) and implies integration of vision and otolithic function (127, 129, 130, 132). These methods are often applied as a solitary line in an otherwise featureless environment (133), but can also be presented with disorienting visual cues such as a tilted frame or rotating disc to evaluate the degree of visual dependence (134–137).

SVV has been found to be a valid and reliable perceptual test and is relatively easy to apply clinically (138–140). Unfortunately, abnormal percepts of verticality are not restricted to vestibular end-organ dysfunction (73, 131, 141, 142), indicating a lack of specificity from a diagnostic perspective. Recently, SVV was suggested to provide prognostic information related to resolution of BPPV (143), and within 6 months of VN errors in SVV has normalized (144).

SVV is most commonly examined in sitting, but can also be performed in standing, see Figure 2 for examples (145). Interestingly, verticality perception is also influenced by both static and dynamic body positioning suggesting integration beyond visual and vestibular contributions (25, 92, 132, 146–151). Importantly, SVV is a distinct construct from subjective body vertical (141). Differences in methodology such as reorienting the line versus indicating the direction of tilt (two-alternative forced choice) as well as whether the subject/patient performs the reorientation actively or halts an observed reorientation make it difficult to compare results across studies and are each prone to specific biases (152). Despite variations in test performance, SVV has excellent validity and reliability (133), thus making SVV testing very clinic appropriate.

#### 3.2.1. Normative data and age effects

The normative values for SVV are less than 2° of error (153–155), and in some studies there is a suggestion that monocular viewing enhances test correlation with disease state (156). Although aging leads to reduced otolith function as assessed by VEMPs, there does not seem to be a consistent adverse effect of aging on *static* SVV accuracy (135, 155). In contrast, older adults make larger errors when tested with a dynamic rotating visual background during *dynamic* SVV testing (135), implying greater deficits in multisensory perceptual integration with age.

#### 3.2.2. Vestibular hypofunction

Individuals with BVH have demonstrated larger visual biases with rod and frame testing and overall greater variability in response accuracy with head on body tilted positions compared to healthy individuals (157). Individuals with bilateral hypofunction, but preserved utricular function based on ocular VEMPs have lower variability and overall higher accuracy for SVV (158) than individuals with unilateral vestibular hypofunction.

### 3.3. Traumatic brain injury

Individuals with TBI often experience dizziness, not necessarily related to vestibular pathology (89, 159–161). It is unclear whether individuals with concussion\TBI experience spatial disorientation as a component of their dizziness, the existing knowledge is more related to navigation and cognitive challenges (12). Brainstem and cortex lesions are associated with larger errors on SVV testing, implicating central pathways are involved in processing these signals (142, 156). Approximately 40% of individuals with non-specific dizziness after a mild TBI demonstrate abnormally large errors on SVV testing (162). It is unclear whether subjective visual vertical abnormalities after TBI are due to peripheral, central or mixed causes reducing diagnostic specificity.

### 3.4. Implications of spatial and verticality testing in rehabilitation

Clinicians interested in including assessments of spatial orientation need to recognize the difference between (1) spatial orientation and (2) spatial updating, and select a test designed to evaluate the desired function. Spatial updating may provide a further window into vestibular cognition, but importantly should also involve progressive improvement due to the inclusion of “ground truth” knowledge of results provided after each movement via verbal feedback or multisensory integration (163–165). This would be characterized by reduction in errors over testing repetitions. For spatial orientation/updating tasks, rotational speed will be important as slower rotation velocities (lower frequencies) will lead to biased and more variable responses. Unlike the expensive equipment necessary for threshold testing, spatial orientation/updating examination can use a barber/salon style chair with a footrest, which may allow for greater clinical uptake. Importantly, spatial performance for individuals with VM and MD is unknown, and whether spatial orientation testing will have added clinical value remains to be determined.

Clinical testing for SVV can be implemented with the low tech “bucket test” (133), which provides widely available access. Virtual reality systems incorporating SVV testing have also become more affordable and widely available (166–169). This increased access combined with high reliability and validity makes testing visual vertical perception an ideal starting place for the clinician that seeks to implement vestibular perceptual testing.

### 3.5. Limitations of spatial and verticality perceptual testing

Currently there is no universal agreement for which style of spatial orientation question holds the most clinical relevance (“where am I” vs. “where was I”), nor is there an idealized response variable, or method for capturing that information (verbal report vs. rotating a dial). Further, the lack of discrimination between unilateral and bilateral vestibular hypofunction in the existing literature may relate to motion profiles below the threshold for semicircular canal inhibitory cut-off (~100°/s), which would allow the intact side to accurately detect motion direction and inertial qualities necessary to derive spatial orientation. The existing knowledge on accuracy ratings for health subjects may reflect testing interval bias (smallest intervals ~30 to 45°). To date, there is no published information regarding test–retest reliability or detectable change values. Due to the variation in application of spatial orientation across studies, clinicians should use and interpret these tests with caution. SVV testing has high reliability and validity; although, the spectrum of disease states that often have abnormally large errors is not restricted to peripheral vestibular disease (156, 170), reducing vestibular specificity. Methods for determining average error after SVV testing are also not consistent across studies with some papers recommending absolute rather than signed errors (171). Additional research is needed to optimize clinical utility for spatial and verticality perception prior to broad clinical adoption.

## 4. Vestibular perception: spatial navigation

Next, we turn our attention to spatial navigation, a complex sensorimotor skill that is required for accurate locomotion within the local (small-scale) and global (large-scale) environments. Egocentric (person-centered) and allocentric (world-centered) orientation processes must be integrated for accurate navigation. This section focuses on relevant data from vestibular-impaired adults who performed real-world spatial navigation tasks. Studies involving only healthy adults, sensory manipulations (e.g., galvanic stimulation), or in which solely virtual navigation was utilized are not included as our emphasis is on findings readily applicable to clinical practice.

Persons with peripheral (40, 74, 172–177), central (178–180), and age-related (181) vestibular dysfunction have impaired spatial navigation. Accuracy in spatial navigation is assessed using path integration tasks, during which a person moves along a specified path or towards a previously viewed target (102). Both linear (40, 172, 175, 176) and geometrically-shaped (182–184) paths have been utilized in prior studies in vestibular-impaired adults. Standardized tests that measure aspects of spatial navigation ability include the Triangle Completion Test (TCT) (182) and the Gait Disorientation Test (GDT) (185). Sense of direction is also commonly measured in studies of path integration, one such measure is the Santa Barbara Sense of Direction Scale (SBSODS) (186).

The TCT (182) is conducted using right triangles, the sides of which are distinctly marked on the floor (Figure 3). Before donning a blindfold and noise-attenuating headphones, participants are instructed to visualize the triangle to be walked. For each triangle, participants are passively guided along one leg, a 90° turn, and then the second leg. Once the examiner lets go, participants are instructed to “turn and complete the triangle” by walking its hypotenuse. The linear distance walked, linear end-point error (distance between where the participant stopped and the origin), and angle error (absolute value of the angular error with respect to the ideal rotation angle) are recorded. Recently, concerns about the reliability of the TCT have been raised by McLaren and colleagues (187) who found that the test–retest reliability for the distance walked was moderate but it was poor for end-point error and angle error.

**Figure 4:**
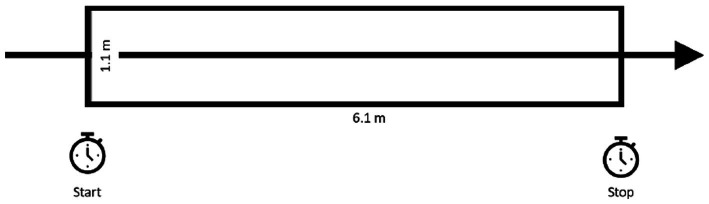
The gait disorientation test (GDT): during the GDT, the participant is timed while walking 6.1 M with their eyes open and again with their eyes closed. The GDT result is the difference in time needed to walk that distance with eyes closed versus with eyes open. GDT scores ≥4.5 s differentiate vestibular-impaired form healthy adults. Figure adapted from Grove et al. (185).

The recently-developed GDT is a less complex navigation task that has excellent test–retest reliability. During the GDT, the participant is timed while walking 6.1 M with their eyes open and again with their eyes closed. Markings on the floor are used to facilitate timing (Figure 4). The result is the difference in time needed to walk that distance with eyes closed versus with eyes open. Although it has not yet been used as an outcome measure, the GDT result is proposed to be a composite measure of several determinants of spatial navigation (e.g., walking speed, spatial memory, and cognitive-perceptual processes) (185).

The SBSODS (186) is a 15-item questionnaire that consists of statements such as, “I have a very good sense of direction”; “I very easily get lost in a new city”; and “I have trouble understanding directions.” Each item is answered using a seven-point Likert scale, and negative statements are reverse-scored. A lower average score (range = 0–7 points) indicates worse self-reported sense of direction. Gandhi and colleagues (188) showed that reduced bilateral saccular function predicted lower scores on the SBSODS in a cohort of community-dwelling healthy older adults. Thus, vestibular afference appears to contribute to one’s perceived sense of direction ability.

### 4.1. Linear small-scale spatial navigation

In one of the earliest studies of the effects of vestibular loss on path integration, Glasauer et al. (189) studied goal-directed linear walking in healthy (*N* = 10) and bilaterally vestibular-impaired (*N* = 7) adults. Participants were tasked with walking straight ahead towards a target anchored to the floor 4 M ahead of them. Walking trials were performed at varying velocities and with either eyes open or while blindfolded. No significant between-group differences were found for distance walked errors; however, vestibular-impaired adults had significantly larger end-point errors. Vestibular-impaired adults also demonstrated significantly greater path curvature during blindfolded walking and significantly slower gait velocity compared with healthy adults. Despite the fact that vestibular-impaired participants were less stable and had larger end-point errors compared with healthy adults, the authors concluded that, because vestibular-impaired adults could perform the blindfolded task, the vestibular system was not necessary for active path integration. Vestibular afference appears to contribute to directional accuracy more so than distance accuracy.

Subsequently, Cohen (172) examined the performance of healthy adults (*N* = 24), individuals with vestibular SWN (*N* = 31), and individuals with chronic (≥3 months duration) peripheral vestibular impairments (*N* = 55) on a straight course. Those with vestibular SWN were tested pre-operatively and at weeks one and three post-operatively. Participants walked 7.62 M, once with eyes open and three times with eyes closed. The time needed to complete each trial, the distance walked before veering, and the angle of veering were recorded for each participant. Compared with healthy controls, SWN patients were significantly impaired on all measures. SWN patients also demonstrated a larger angle of veering and longer duration of trials compared to controls. Pre-operatively, SWN patients were significantly impaired compared to controls for the distance walked before veering. Compared with the pre-operative status, SWN patients were more impaired 1 week post-operatively for the angle of veering. Partial recovery of path integration ability was evident at week three post-operatively as individuals with SWN walked further before veering, veered less, and required less time to complete the task compared to week one post-operatively. These findings conflict with Glassauer et al. (189); however, differences in the length of the course may explain this discrepancy. Importantly, Cohen’s results suggest that central vestibular compensation partially mitigates path integration deficits following vestibular deafferentation.

### 4.2. Complex small-scale spatial navigation

Peruch et al. (40) evaluated spatial performance in unilaterally vestibular-impaired (*N* = 8) and healthy adults (*N* = 6) who were assessed on visual and non-visual navigation tasks. Performance was assessed at four time points before and at 1 week, 1 month, and 3 months after impaired participants underwent unilateral vestibular neurotomy. Healthy adults were assessed at similar time intervals. Participants first explored an environment in which four locations were marked by different objects. Then, participants attempted to navigate to those locations by reproducing the routes that were traversed during exploration, by reversing routes, or by taking shortcuts (making spatial inferences). Finally, participants performed identical navigation tasks in virtual reality while seated in front of a large projection screen. Navigation in the virtual environment was controlled by the participant using a joystick. Prior to surgery, vestibular-impaired participants had lower angle errors during real-world navigation when reproducing previously traversed routes compared with healthy controls. The authors postulated that patients were using compensatory strategies that relied on somatosensory afference prior to surgery to learn and reproduce routes. However, since no between-group differences were found for navigating with shortcuts prior to surgery, compensatory mechanisms were insufficient for higher-level spatial processing. Angle errors were also larger for patients compared to controls during virtual navigation, which suggests that vestibular afference must be integrated with visual cues for accurate navigation. Angle errors in real-world and virtual navigation increased after surgery for patients; however, between-group differences were seen only at 1 week post-operatively. Thus, in agreement with Cohen (172), central vestibular compensatory mechanisms may result in rapid restoration of spatial navigation ability following vestibular deafferentation.

Glasauer et al. (94) investigated the effects of vestibular dysfunction on complex path integration ability in healthy (*N* = 7) and vestibular-impaired (*N* = 5) adults. Vestibular-impaired participants had chronic (several months duration) symptoms and were assessed after the completion of vestibular rehabilitation. After viewing the course, participants walked a right triangular path (each leg length = 3 M). The task was completed in both the clockwise and counter-clockwise directions three times while blindfolded, then once with eyes open. Three-dimensional trajectories in 6° of freedom were collected from head-worn markers. The distance walked was similar to the required distance in both groups and there were no between-group differences. Additionally, there were no significant between-group differences in the arrival error at the first corner; however, there were significant between-group differences for the end-point error. Larger end-point errors in vestibular-impaired participants were attributed to angle errors. The authors postulated accuracy in path integration depends on proprioceptive and motor efference information related to turning corners and is enhanced by sensory input regarding angular velocity from the vestibular system. Interestingly, unilaterally and bilaterally vestibular-impaired participants had increased angular errors, and unilaterally involved participants had larger errors when turning towards their uninvolved side. The reason for this is not immediately clear, but larger variability in the lead time between the predictive head turn in advance of the turn may have been a factor for individuals with vestibular-impairments. Additionally, differences in walking and head turning velocities between the smaller first turn and the larger second turn may have influenced performance.

Péruch et al. (184) assessed path integration in unilaterally vestibular-impaired adults (*N* = 7). Participants were assessed 1 day prior, as well as 1 week and 1 month after unilateral vestibular neurectomy. Healthy, age-matched controls were also assessed at the same intervals. All participants were tasked with either (1) reproducing a right triangular path (leg length = 3 M) that had been walked with the examiner, (2) completing the reverse of the route walked with the examiner, or (3) taking a shortcut back to the origin. These tasks were performed in a real-world environment (while blindfolded) and in virtual reality (head-mounted display). Absolute and signed turn error, as well as absolute and signed distance error were measured. Unilateral vestibular deafferentation impaired the orientation component of spatial navigation, and the extent of these impairments differed depending on task complexity and on which sensory cues are available for interpretation. Performance on tasks with greater complexity significantly differed between healthy and vestibular-impaired adults. When performing more complex spatial navigation tasks, vestibular-impaired participants also showed greater distance errors than healthy controls. From these data, one can conclude that task complexity and which sensory inputs are available during performance are likely relevant for training path integration.

Guidetti et al. (174) assessed whether spatial navigation deficits persisted in adults with well-compensated UVH. In this study, vestibular-impaired (*N* = 50) and healthy (*N* = 50) adults were tasked with visually memorizing three different routes marked on the floor (a triangle, a circle, and a square), and, then, to walk these routes while blindfolded and again with vision. The time to complete each task was recorded. Additionally, participants completed a test of short-term visual–spatial memory and neuropsychological testing. Even well-compensated adults with vestibular-impairment required more time to complete the tasks with eyes closed compared with controls; however, there were no within-group differences in completion time for clockwise and counter-clockwise navigation. Vestibular-impaired participants also had worse visual–spatial ability and higher levels of anxiety and depression, which may have influenced spatial navigation performance.

Xie et al. (181) reported on the effects of age-related vestibular decline on spatial navigation ability. A total of 48 adults participated, including young healthy controls (*N* = 9), older healthy controls (*N* = 15), and older adults with dizziness (*N* = 24). All participants were tested for vestibular function using cervical VEMPs and the video head impulse test, they also completed the TCT. Results showed a step-wise increase in end-point errors from young controls to older controls to older participants with dizziness. Additionally, both control groups had smaller angle errors than the group with dizziness. End-point and angular errors were increased for participants (control or dizzy) with abnormal otolith and semicircular canal function. Thus, even sub-clinical changes in vestibular function may affect spatial navigation.

### 4.3. Complex large-scale navigation

Schoberl et al. (190) used a novel paradigm to assess real-world spatial navigation performance in adults with either complete or incomplete bilateral vestibular paresis and healthy matched controls. All participants performed standardized navigation tasks in an outpatient clinic. After a familiarization phase, the examiner cued participants to find specific landmarks without guidance and in particular sequences that required participants to retrace a familiar route, traverse a new route, or to take shortcuts. Ten minutes were allowed for the completion of 15 routes; if a participant could not locate a specific landmark, the examiner gave instructions to find the next item. The primary outcome was the error rate, defined as not being able to find, passing by, or ignoring a specific item. Gait velocity, saccades, and visual fixations were also recorded using a mobile eye tracking device. Adults with BVH\L performed worse than controls when navigating new routes, but there were no between-group differences in errors when retracing familiar routes. The number of errors was associated with the extent of vestibular loss. Additionally, compared to controls, adults with BVH\L had greater fluctuations in gait speed, spent less time at intersections, and utilized shortcuts less often. Further, adults with BVH\L had fewer gaze fixations and made horizontal head movements less often while traversing new routes compared with controls. Thus, persons with BVH\L may have more difficulty navigating and may report more disorientation in unfamiliar environments compared with familiar environments.

Dordevic et al. (191) studied cognition, spatial skills, and path integration in adults with unilateral or bilateral vestibular hypofunction (*N* = 15) and healthy matched controls (*N* = 15). All participants completed clinical vestibular tests of balance, the video head impulse test, caloric tests, the TCT, and whole-body rotational memory. Tests of visuo-constructive, spatial, attention, and concentration abilities were also administered using paper and pencil. Additionally, participants underwent imaging for structural brain analyses. Adults with UVH\BVH performed worse than controls on all clinical balance tests, whether these were conducted with eyes open or closed. Compared with controls, the impaired participants made greater distance errors on the TCT when passively transported in a wheelchair, and demonstrated worse rotational memory. However, there were no significant between-group differences in visuo-spatial, general cognitive abilities, and whole-brain or region-of-interest gray matter volumes. Although these results suggest that visuo-spatial and general cognitive abilities are preserved in persons with impaired vestibular cognition, a recent systematic review (192) provides summary evidence of the effects of vestibular loss on cognitive function.

Biju et al. (193) investigated the effects of vestibular deficits on route-based and place-based navigation in real-world and virtual environments. Adults with unilateral or bilateral vestibular hypofunction (*N* = 20) and matched controls (*N* = 20) navigated two routes in two environments, an outpatient clinic and virtual reality on a laptop computer. Prior to traversing the real-world routes, participants were familiarized with the route and specific landmarks, placed in a wheelchair and blindfolded, rotated around several times to disorient them, and, then, passively transported to the start via a random route. In the route-based task, participants were instructed to reproduce the route traversed during familiarization. During the place-based task, participants were asked to navigate to the landmark at the end of the route using the shortest path possible. Participants also completed a judgement of relative direction task in the real-world and on the laptop, as well as the SBSODS. Vestibular-impaired participants had higher path length ratios (required versus actual path) than controls. Path ratios were higher in the virtual compared to the real environment. Additionally, the place-based task resulted in higher path ratios compared with the route-based task. Regarding judgements of relative direction, there were no significant between-group differences; however, errors were greater in the virtual compared to the real environment. Although there was significant correlation between performance in real and virtual environments for controls, no such associations were found for vestibular-impaired participants. Additionally, no between-group differences were found for self-reported sense of direction. Together, these findings suggest that vestibular afference contributes to both route-based and place-based spatial navigation. Additionally, the authors postulated that, although healthy adults likely used the same strategies to navigate in real and virtual environments, vestibular-impaired participants employed different navigational strategies.

### 4.4. Spatial navigation training

Cohen and Kimball (194) were the first to assess whether vestibular rehabilitation leads to improvements in spatial navigation. These authors assessed path integration in adults with chronic peripheral vestibulopathies (*N* = 53) who completed 4 weeks of vestibular habituation exercises. In this study, the path integration task was the same as used by Cohen (172), described above. Performance was assessed pre-and post-rehabilitation. Gait velocity increased and the angle of veering decreased over time. Natural recovery is an unlikely confounder since all participants had reported brief episodes of vertigo elicited by head movement for at least 2 months prior to enrollment. The authors concluded that performing exercises that create visual-vestibular interaction leads to reduced spatial disorientation. Thus, one may not need to practice path integration tasks to achieve improvements in this domain.

Ishikawa and Zhou (195) assessed whether spatial navigation could be improved in a group of adults with self-reported poor sense of direction (*N* = 40) based on the SBSODS (186). Participants were assigned to perform path integration training with knowledge of results feedback, with (Group 1) or without (Group 2) allocentric training. The performance of these two groups was compared to that of a cohort of adults with average self-reported sense of direction. A test of mental rotation was also administered. All participants completed six weekly sessions in which they were tasked with actively walking one triangular, one quadratic, and one crossing path while blindfolded. Prior to traversing each path twice, participants who received allocentric training oriented to cardinal directions (e.g., north). Once they thought they had reached the origin, participants’ perceived location and orientation was recorded; then, participants received feedback about their accuracy. During each study visit, all participants in both groups also traversed one of six real-world routes in a residential district in Tokyo, Japan that were 450 M long and incorporated five turns and four easily recognizable landmarks. After completing the real-world route, participants were assessed for their accuracy in estimating the orientation of one landmark relative to another and the distance between sets of landmarks. The groups did not differ in mental rotation or path integration at the outset. Allocentric training improved the accuracy of direction estimates, and feedback alone led to improved accuracy in straight-line distance estimates and sketching route maps. Thus, translation between egocentric and allocentric representations is difficult and may not be readily trainable.

### 4.5. Implications for rehabilitation

The works reviewed herein lead to several conclusions. Interventions aimed at reducing misperceptions (non-specific dizziness/vertigo), like habituation, gaze stability training, and balance/gait activities (196), may lead to improvements in spatial navigation without task-specific training of path integration for individuals with vestibular hypofunction. Navigation training will likely produce task and/or environment specific effects; thus, the type of training should be individualized to address each patient’s specific deficits. Baseline cognitive ability and emotional state may influence navigational rehabilitation outcomes. The optimal training parameters for interventions aimed at improving path integration skills are not yet known; however, intensive training appears to facilitate robust improvements immediately post-training. Practicing path integration using routes of varying levels of difficulty and incorporating varying numbers of turns and path crossings may be a useful intervention, particularly if feedback regarding allocentric orientation is provided.

## 5. Vestibular perception: vestibular cognition

The domains of cognitive performance are not independent of each other. Within each domain there are often subdomains which refer to component cognitive abilities or processes. To fully understand a patient’s clinical presentation related to vestibular perception it is critical to consider the distinct contribution of all cognitive abilities or impairments. One challenge in synthesizing the literature related to the interplay between vestibular and cognitive function is the variability in the assessment methods used across investigations. Another challenge is discerning if multiple cognitive abilities are contributing to the cognitive performance and/or if the specific assessment method is truly measuring the domain that it was intended to assess.

It is important for clinicians to understand which domain of cognition is impaired because it could help determine specific interventions to include within the plan of care and/or determine how best to modify the delivery of vestibular interventions to optimize outcomes for their patients. Unfortunately, the best cognitive assessment methods for vestibular scientists and clinicians to use to measure each of the component cognitive abilities/processes is yet to be determined. Despite the discordance in assessment methods used, there is considerable evidence from human studies suggesting that multiple domains and subdomains of cognition (including: attention; executive function; memory; and visual spatial ability) are associated with vestibular disorders (102).

The findings related to cognitive processes that have been observed in investigations of common vestibular diagnoses including UVH, BVH, VM, and MD will be illustrated below. The specific assessment method for each finding will be indicated within parentheses following the cognitive ability/impairment. While navigation is considered a visual spatial ability, it was discussed separately in the previous section.

### 5.1. Unilateral vestibular hypofunction

Compared to healthy controls, some studies show that people with UVH display impaired *attention, memory*, and *visual spatial ability*. However, there are conflicting findings which could be due in part to the choice of assessment methods used and/or the specific component processes being assessed. A 2022 study reported that compared patients with healthy controls, people with UVH do not differ significantly in working memory abilities (digit span test), executive function (Stroop Task), or attention (Attention Network Test and Flanker Test) (197).

Attention impairments were however reported in adults with UVH (*n* = 15) using a dual-task paradigm that measured concurrent postural demands and cognitive tasks (reaction time) (198). Redfern et al. found that during concurrent balance or vestibular tasks people with UVH displayed a delayed response and decreased accuracy for the cognitive task when the postural or vestibular challenge was added. Another study also found that people with UVH (*N* = 14) also displayed decreased cognitive performance during dual-task gait challenges compared to healthy controls (199). Both investigations yielded similar conclusions suggesting that the brain prioritizes attentional resources to maintain balance and motor tasks at the expense of other cognitive tasks.

Visuospatial ability impairments (visual scanning reaction time test and processing speed tasks) have been reported in people with UVH (*N* = 16) when compared to healthy controls (200). A 2020 investigation of people with acute UVH (*N* = 21) also reported impaired visuospatial ability (Benton’s Judgment of Line Orientation test), attention and psychomotor speed (verbal and non-verbal cancellation tests), and short-term memory and mental manipulation (backward digit span) when compared to healthy controls (201).

Conversely, two other studies found no differences in visuospatial cognitive processes. Spatial perception (SVV including rod and frame conditions) (124), spatial memory also did not differ and the hippocampal volume did not significantly differ in people with UVH (202). Another study of people with acute UVH (*N* = 28) showed that visuo-spatial ability (line bi-section task, Albert’s test, Bell’s test, and a figure copying task) was not clinically impaired (203).

### 5.2. Bilateral vestibular hypofunction

Compared to healthy controls, people with BVH have been shown to have impaired *attention, executive function, memory*, and *visual spatial ability*. It is appreciated that cognitive impairments are typically more pronounced in BVH compared to UVH (200).

A recent 2022 study reported clinically meaningful lower scores for global cognition (RBANS-H total scale) in people with BVH (*N* = 34) compared to healthy controls (*N* = 34) (204). Those authors reported that the subdomains of memory, visuospatial cognition, and attention on the RBANS-H assessment were impaired in BVH but there were no differences observed with language or delayed memory subdomains (204). Attention impairment has also been demonstrated using a walking and cognitive dual task paradigm, whereby both walking and cognitive performance was degraded for people with BVH (*N* = 12) compared to healthy controls (*N* = 12) (205).

Another investigation revealed that people with BVH (*N* = 18) had impaired short-term memory (Theory of Visual Attention), executive function (Backward Corsi Block Tapping test), processing speed (Stroop Color-Word test), and visuospatial abilities (visual scanning reaction time) compared to people with UVH (*N* = 16) and healthy controls (*N* = 17) (200).

Significant spatial memory deficits (computerized virtual water Morris maze task) associated with bilateral hippocampal atrophy have been observed in people with BVH (173, 177, 206). While there was not a difference between healthy controls and people with UVH, investigators of another study reported that people with BVH have worse spatial imagery (mental body transformations) (124).

### 5.3. Vestibular migraine and Meniere’s disease

There is conflicting evidence regarding cognitive impairment in people with MD. A 2023 investigation of cognition using the Montreal Cognitive Assessment (MoCA) scale people with MD (*N* = 30) and healthy controls (*N* = 17) found significantly lower memory (MoCA memory sub score) and overall cognition (MoCA total score) in the people with MD (207). Conversely, another 2023 study concluded that neither MD (*N* = 19) or VM (*N* = 19) have significant differences in cognition compared to healthy controls (208). The domains of cognition assessed in the study of people with VM and MD included: global mental status (Mini Mental State Examination); working memory and attention (Stroop test); spatial cognition (Benton’s Judgement of Line Orientation test); and working memory, visual processing, visuospatial skills, attention, and processing speed (Trail Making Test) (208). The lack of differences could be due to the episodic nature of these diagnoses and confounding accompanying symptoms that are associated with VM and MD and/or the choice of the assessment method used to measure cognition.

### 5.4. Implications for rehabilitation

Notably, there are many factors that can impact cognition in people with vestibular disorders including acuity of the vestibular diagnosis, age, anxiety, depression, hearing, medications, sleep hygiene. Additionally, poorer self-reported sense of direction has been shown to be significantly associated with vestibular impairment (188). Regardless of the cause of the interplay between cognitive and vestibular function seen in common vestibular diagnoses which has been proposed in the literature (209, 210), appreciating the common co-occurrence of cognitive and vestibular impairments could help clinicians develop therapeutic strategies to facilitate recovery.

The impact of cognitive impairment on vestibular rehabilitation outcomes is not well understood. Although cognitive impairment may not alter the physiology of vestibular compensation, it could impact recovery if it interferes with a person’s ability to comply with exercises and recall what he/she has previously learned in therapy. Micarelli et al. suggested worse long-term function in people with cognitive impairment (211). A modification for the delivery of vestibular rehabilitation that is in alignment with clinical practice guidelines has been published that emphasizes motor learning abilities for people with cognitive impairment (212). Utilizing modified vestibular therapy interventions is currently being investigated in people with Alzheimer’s disease (213).

Future work should aim to determine the best clinically feasible assessment methods to screen and assess cognition for people with vestibular disorders. It is proposed that working memory should be assessed in people with peripheral vestibular disorders (214). An important clinically relevant conclusion from a 2023 dual task investigation during functional gait performance in people with chronic vestibular disorders reiterated the importance of measuring cognition in people with vestibular disorders to ensure the inclusion of appropriate interventions (215). Identifying the specific cognitive abilities/processes that are impaired will allow development of targeted interventions to optimize outcomes for people with vestibular disorders.

## 6. Summary and conclusions

As of this writing, few studies have examined the clinical application of vestibular perception in any form despite the extensive literature demonstrating disease state discrimination. Threshold testing in current form has limited clinical availability primarily due to the equipment and space requirement. Despite this limitation, roll thresholds are associated with postural control and future development of more clinic friendly perceptual assessments is needed. Spatial orientation and navigation testing may have greater clinical utility, but current testing methods have either unclear or low test–retest reliability. Screening for neurocognitive function and other forms of vestibular function has a place in vestibular rehabilitation, but additional research to determine clinical validity and reliability is needed to expand integration beyond limited use for initial clinical screening. Additional work is also needed to characterize the exercise interventions that optimally improve perceptual deficits for individuals with vestibular disease.

## Ethics statement

Written informed consent was obtained from the individual(s) for the publication of any identifiable images or data included in this article.

## Author contributions

CG: Funding acquisition, Methodology, Writing – original draft, Writing – review & editing. BK: Funding acquisition, Methodology, Writing – original draft, Writing – review & editing. AW: Funding acquisition, Methodology, Writing – original draft, Writing – review & editing. EA: Conceptualization, Funding acquisition, Methodology, Supervision, Writing – original draft, Writing – review & editing.
